# Important functional distress in a teenager with optic nerve drusen

**Published:** 2019

**Authors:** Alina Simona Lazar, Simona Stanca, T. Horia Stanca

**Affiliations:** * “Prof. Dr. Agrippa Ionescu” Clinical Emergency Hospital, Bucharest, Romania; ** “Carol Davila” University of Medicine and Pharmacy, Bucharest, Romania; *** Pediatric Clinic, “Grigore Alexandrescu” Children Emergency Hospital, Bucharest, Romania

**Keywords:** optic nerve drusen, pseudopapilledema, optic nerve pathology, visual field defects, perimetry

## Abstract

We present a case of bilateral optic disc drusen and severe visual field loss in a female patient diagnosed at a very young age.

## Introduction

Optic nerve head drusen is a congenital condition of the optic disc. It consists of deposits of calcium, amino acids, nucleic acids, mucopolysaccharides, and iron, accumulated in the substance of the optic nerve, anterior to the lamina cribrosa [**1**]. 

The etymology of the word drusen derives from the German word druse, used in geology to describe a rock that contains a cavity lined with crystalline incrustations [**1**]. 

Optic nerve drusen may appear sporadically, or in some cases genetically, with an autosomal dominant pattern of inheritance [**2**]. 

It is the most common cause of pseudopapilledema, being very important to be distinguished from true papilledema [**3**].

There are two types of optic nerve drusen. The first one is buried drusen, deep and hidden under the disc surface. Buried drusen gradually evolves to exposed drusen, which is superficial and directly visible at the disc surface [**4**].

Optic nerve drusen is usually asymptomatic and discovered incidentally during a routine ophthalmological examination. The central visual acuity is in general well preserved. However, optic nerve drusen is associated with peripheral visual loss and patients present with visual field defects that progress over time [**5**]. 

Currently, there is no treatment available against the anatomical and functional changes induced by the presence of drusen in the optic nerve. 

## Case report

We present the case of a 13-year-old female patient, known with severe visual field loss, who referred for another opinion regarding the ophthalmological diagnosis. 

Anamnesis at presentation revealed that at the age of 9 years and 3 months, on a routine ophthalmological examination, papillary calcification and retinal hemorrhage were discovered in the left eye. At that moment, the suspicion of intracranial calcifications was raised. The patient underwent clinical neurological examination, EEG, and cerebral MRI, all of them revealing a normal aspect. The patient was also recommended fluorescein angiography, which showed papillary autofluorescence. The diagnosis established then was papillary drusen in both eyes, buried in the right eye and mixed in the left eye and the patient was recommended to keep it under observation, together with a periodical examination of the visual field. 

The patient had had multiple examinations of the visual field over the time. 

The first visual field examination of the right eye showed an arcuate nasal defect, in the superior nasal quadrant, sketching an aspect of nasal step, structure of the sensitivity defect that in 3 years time evolved into a quadranopsia. 

In the left eye, the first visual field examination showed inferior nasal quadranopsia, extended superiorly with a nasal arcuate defect respecting 20° centrally, which after three years evolved into a paracentral diffuse defect with an island of central vision of 5°. 

However, over the time, the examination of the visual field was made with different types of machines, and no correlation of the modifications could be made objectively. 

The patient received several different diagnoses from several different ophthalmologists, among which optic nerve drusen; papillary oedema and hamartoma have to be mentioned. 

At presentation, the patient’s visual acuity was 20/ 20 with correction for the RE and 20/ 20 without correction for the LE, with a refraction ROD: -1 DSf<> -0.75 DCyl, 179* and ROS: +0.50 DSf<>-0.75 DCyl, 167* and a cycloplegic refraction: OD: -0.75 DSf<> -1 DCyl, 168*, OS: +0.75 DSf<> -1 DCyl, 170*.

The intraocular pressure was 19 mmHg GAT in the right eye and 13 mmHg GAT in the left eye. 

Slit lamp examination of the anterior segment revealed no pathological changes for both eyes, and the red-discrimination test was also normal.

Fundoscopy presented only with papillary pathological modifications. 

**Fig. 1:**
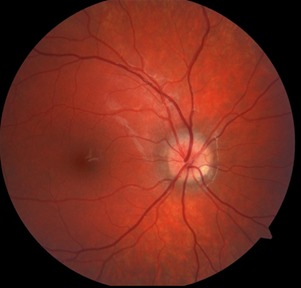
Fundus photography of the right eye

The optic disc in the right eye was elevated, with relatively clear margins, pink color, and the absence of cupping. At 5 o’clock meridian, a nodular yellow mass, with irregular outline, could be noticed (**Fig. 1**). 

**Fig. 2:**
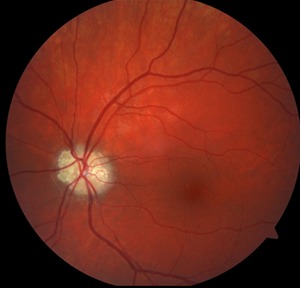
Fundus photography of the left eye

In the left eye, the optic disc was also elevated, pale, of irregular outline, and the absence of cupping was noticed. Nodular, yellow, reflective protrusions, with irregular contour and brambleberry shape could be noticed (**Fig. 2**). 

The retinal vessels, the macula, and the retinal periphery presented no pathological changes in either of the eyes.

The clinical examination suggested the diagnosis of optic nerve drusen in both eyes. B-scan ultrasonography and optical coherence tomography (OCT) examinations were used for the confirmation of the diagnosis.

B-scan ultrasonography is considered the gold standard method for the detection of optic disc drusen. In this patient’s case, it showed round, hyperechoic structures, observed at the optic nerves of both eyes. The A-scan mode, which was overlapped on the structure only for the left eye, showed hyperreflectivity at the anterior side of the optic nerve, of supraretinal intensity. 

**Fig. 3:**
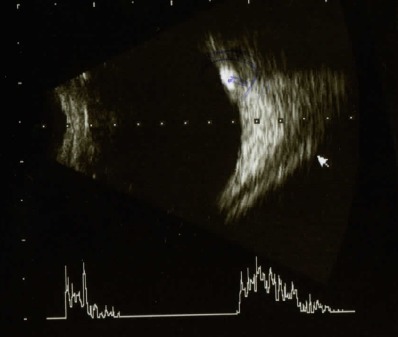
Ultrasonography right eye

**Fig. 4:**
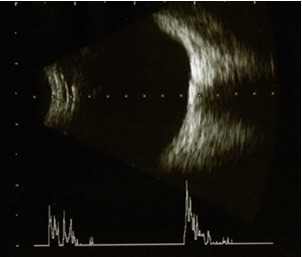
Ultrasonography left eye

Optical coherence tomography is a useful examination in the assessment of the structure and the anatomical shape of the drusen, and in the analysis of retinal nerve fiber layer (RNFL) and GCL-IPL complex. 

For patients under 18 years old, however, there is no normative database regarding the normal values of the analyzed parameters, therefore these analyses are useful only for patient’s follow-ups. 

**Fig. 5:**
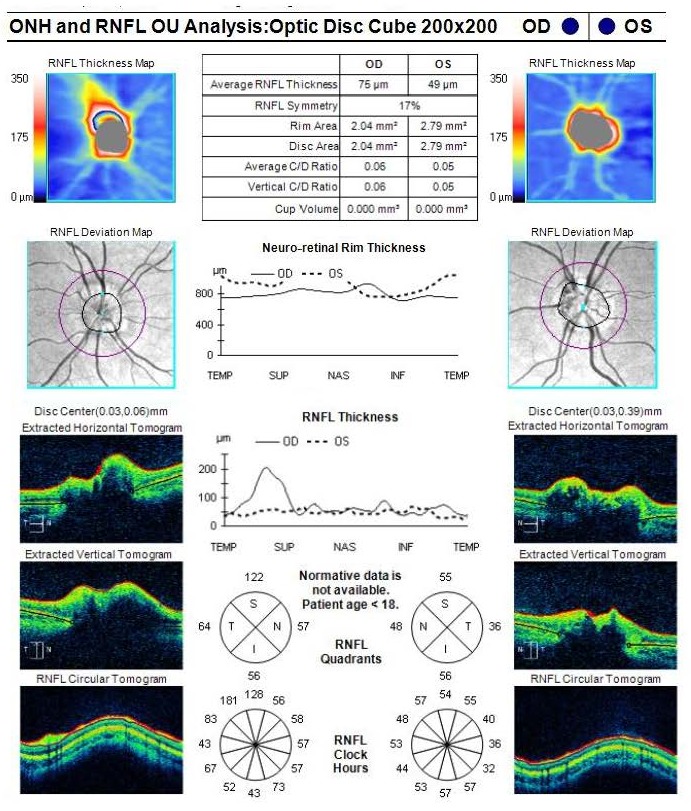
Optical coherence tomography of the optic nerve in both eyes

The OCT scan of the optic nerve showed a prominent aspect of the optic disc, with a lower value of average RNFL thickness in the left eye compared to the right eye (**Fig. 5**).

**Fig. 6:**
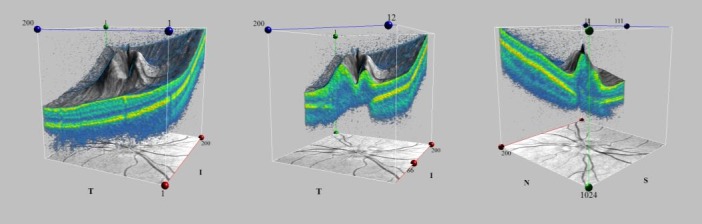
Optical coherence tomography 3D Visualization of the Optic Disc Cube of the right eye

**Fig. 7:**
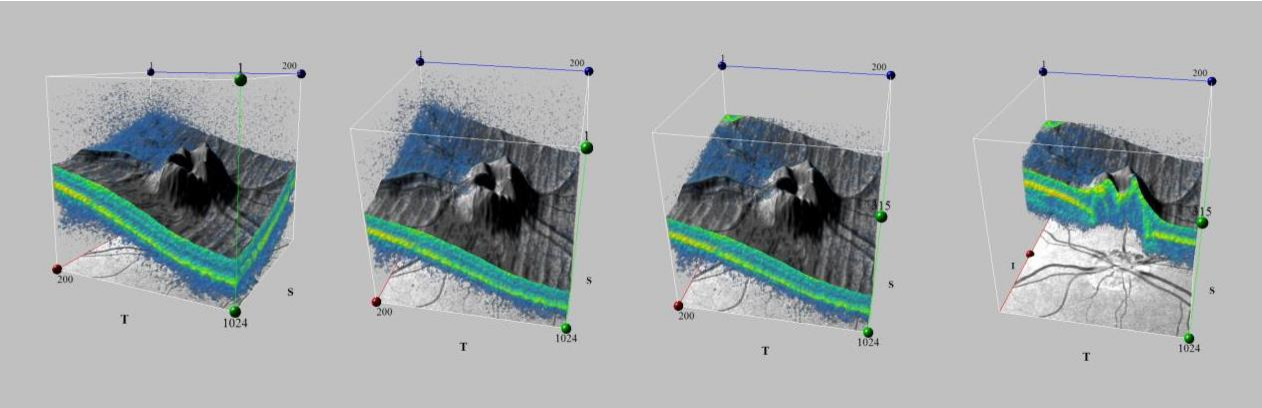
Optical coherence tomography 3D Visualization of the Optic Disc Cube of the left eye

**Fig. 8:**
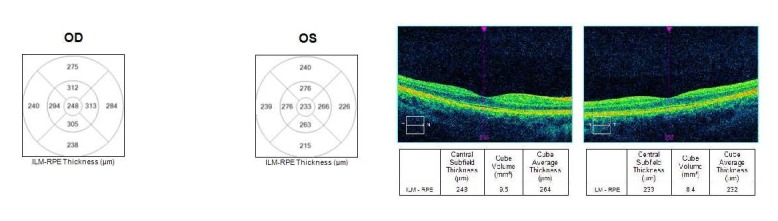
Optical coherence tomography macular thickness analysis for the right and left eye

Macula was structurally normal, with an asymmetry of macular thickness, thinner in the left eye, compared to the right eye (**Fig. 8**).

**Fig. 9:**
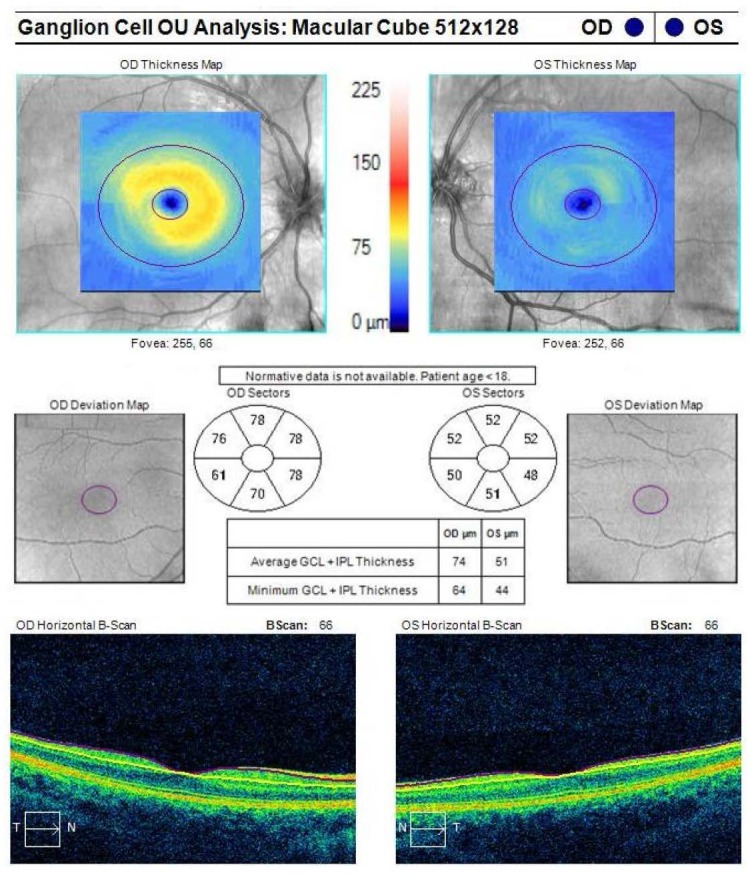
Optical coherence tomography ganglion cell layer analysis

There was also an asymmetry of thickness regarding the GCL-IPL complex, which was thinner in the left eye compared to the right eye (**Fig. 9**). 

The investigations confirmed the diagnosis of optic nerve drusen in both eyes.

The differential diagnosis in the case of this patient took into consideration the following pathologies: 

• Papilloedema – excluded by B-scan ultrasound;

• The existence of an intracranial expansive process – excluded by clinical and imagistic examinations;

• Optic nerve tumors 

o Astrocytic hamartoma – the proliferation of astrocytic cells occurs above the optic disc, whereas optic disc drusen is located in the substance of the optic nerve. 

o Optic nerve sheath meningioma – excluded by clinical and imagistic examinations.

• Leber optic neuropathy – it typically presents with severe loss of central vision.

• Infiltration of the optic nerve (leukemia, lymphoma) – excluded by normal laboratory tests.

**Fig. 10:**
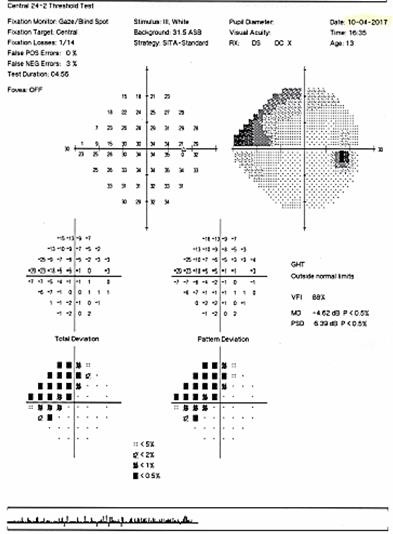
Humphrey visual field of the right eye at presentation showing a superior nasal altitudinal scotoma

**Fig. 11:**
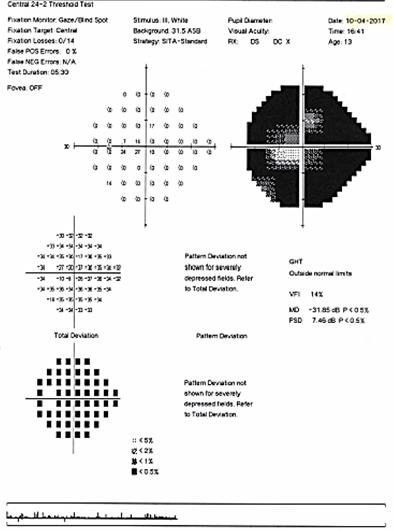
Humphrey visual field of the left eye at presentation showing an important constriction of the visual field, with the preservation of an island of central vision

The patient’s visual field examination at presentation revealed a superior nasal altitudinal scotoma at the right eye (**Fig. 10**), and at the left eye an important constriction of the visual field, with the preservation of a small 15* island of temporal paracentral vision (**Fig. 11**).

The patient was not recommended any treatment, but only periodical follow-up with visual field examination at every 4-6 months, and annual OCT. 

**Fig. 12:**
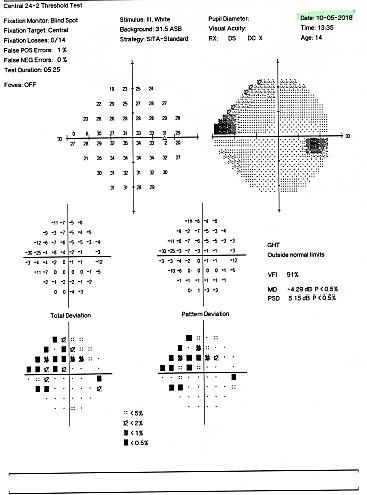
Humphrey visual field of the right eye at follow-up one year later

**Fig. 13:**
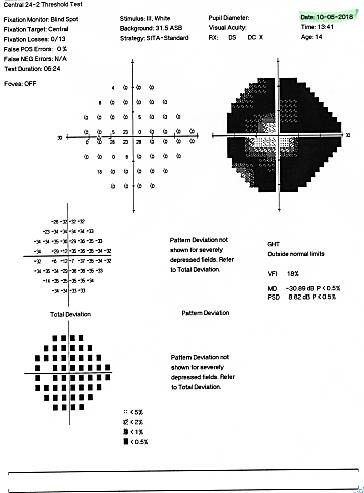
Humphrey visual field of the left eye at follow up one year later

The patient came back a year later for follow-up. At examination, there was no progression of the visual field alterations (**Fig. 12**,**13**), but the intraocular pressure was at the superior level of the normal range, 21 mmHg GAT for the right eye and 20 mmHg GAT for the left eye.

Therefore, the patient was recommended the treatment with a prostaglandin analogue to prevent the exacerbation of the visual field loss in order to attenuate the mechanical compression on the ganglion cells axons and to improve the blood flow to the optic nerve head. 

## Discussion

Optic nerve drusen can be diagnosed in patients of all ages. The prevalence of optic nerve drusen reported in children was of 0.4% [**6**], whereas in adults was of 0.5% to 2.4% [**7**]. 

In children, drusen is usually buried, and difficult to visualize. The mean age reported in literature for the appearance of the superficial component of optic nerve drusen is 12 years old [**8**]. In this patient’s case, drusen became visible in the left eye at a much younger age, of 9 years and 3 months. 

Optic nerve drusen are usually bilateral [**5**] and affect both male and female patients equally [**1**].

The physiopathology of optic nerve drusen is incompletely understood. It is thought to be the consequence of a slow degenerative axonal process, having a genetic basis. 

Multiple theories are taken into account.

One theory suggests that optic nerve drusen is the consequence of axonal metabolism alteration, with the deposition of calcium into the mitochondria and the extrusion of mitochondria into the extracellular space. The consequent development of optic nerve drusen is the result of the continuous calcification process of the extruded mitochondria that leads to the formation of small-calcified bodies, which fuse anterior to lamina cribrosa [**9**]. 

Another theory regarding the development of optic nerve drusen is the association with an anatomically smaller diameter of the scleral canal. According to this theory, small scleral canals exert mechanical compression on the nerve fibers, affecting the axoplasmic flow. Consequently, as axonal degeneration produces, mitochondrial extrusion, and calcification lead to the development of optic nerve drusen [**10**-**12**]. However, there are studies that found no correlation between the scleral canal size and optic nerve drusen, therefore further investigation is needed to be conducted on this theory [**13**].

It was also postulated that optic nerve drusen is associated with a congenital dysplasia of the optic disc and a congenitally abnormal optic nerve vasculature pattern [**2**]. 

All of the factors mentioned above are thought to have a genetic basis, but the gene involved is unidentified [**2**]. 

There are several theories regarding the pathogenesis of visual field defects in patients with optic nerve drusen. 

A connection was made between the visual field loss and the impairment of the axonal transport due to a smaller size of the scleral canal in eyes with optic nerve drusen [**14**].

The pathogenesis of visual field defects in patients with optic nerve drusen was also linked to the mechanical compression exerted by the calcified bodies on the ganglion cell axons, with axonal degeneration and cellular death [**15**]. 

Another mechanism proposed for the appearance of visual field defects was the ischemia of the optic nerve head due to lower systolic flow velocities in the central retinal artery in patients with optic nerve drusen [**16**]. 

The types of visual field defects described in association with optic nerve drusen were nerve fiber bundle defects, arcuate defects, enlargement of the blind spot and concentric narrowing of the visual field [**1**]. 

Even though visual field defects may appear in both types of drusen, they were associated with a higher prevalence in the eyes with superficial drusen, rather than in those with buried drusen [**17**]. 

The mean age reported in literature for the discovery of visual field loss is 14 years old [**8**]. In this patient’s case, the visual field loss was discovered at a much younger age – 9 years and 3 months. 

Visual field defects slowly progress over time, in accordance to the appearance and evolution of the optic nerve head drusen, usually with the patient being asymptomatic [**18**]. 

There are studies suggesting that the most accelerated functional loss occurs during adolescence, corresponding to the period of transition from buried disc drusen to superficial optic nerve drusen, followed by a period with minimal or no alterations anatomically and functionally, during adulthood [**19**]. However, further studies need to be conducted in this regard. 

A modality reported to assess the progression of optic nerve drusen is the measurement of RNFL and GCL-IPL complex thinning by means of optical coherence tomography. 

It was found that eyes with optic nerve head drusen present with significant thinning of the RNFL and GCL-IPL complex measured by optical coherence tomography, and that GCL-IPL complex thinning might be an earlier indicator of cellular loss, before the appearance of RNFL thinning, in eyes with buried drusen [**20**]. 

Also, in another study, mean peripapillary RNFL thinning was significantly correlated with visual field defects as measured by perimetric mean deviation. The same study also showed that greater peripapillary RNFL thinning as well as visual field defects are found in eyes with superficial optic nerve drusen, compared to eyes with buried drusen [**21**].

A correspondence between RNFL thinning measured by OCT, the optic disc sector in which drusen aggregated the most and visual field defects was also reported [**22**]. 

Given the fact that there is no established method which best assesses the progression of optic nerve head drusen, more studies on this topic are necessary. 

There is currently no treatment accepted against the visual loss caused by optic nerve head drusen. 

In the case of patients with optic nerve head drusen and borderline intraocular pressure, the reduction of intraocular pressure with intraocular pressure lowering drops has been proposed, in an attempt to reduce the mechanical compression on the ganglion cells axons [**5**,**23**].

The use of Gingko biloba as a neuroprotective agent was suggested to have beneficial effects in patients with normal tension glaucoma, but further studies are necessary with respect to visual field preservation in patients with optic nerve drusen [**24**].

A surgical treatment was also proposed for the treatment of visual field loss in patients with optic nerve drusen. Radial optic neurotomy was reported to be successful in a number of patients with optic nerve drusen, by reducing the pressure on the optic nerve at the scleral outlet [**25**-**27**]. However, more studies on the surgical treatment are necessary, given the questionable aspects regarding the safety and efficacy of the procedure. 

In general, the prognosis is reported to be good for patients with optic nerve drusen, with the reduction in the peripheral vision, but the preservation of the central vision. One study reported that the worst visual acuity for patients with optic nerve head drusen was 20/ 50 [**28**]. 

Severe loss of visual acuity in patients with optic nerve drusen is commonly associated with acute complications.

The most frequent cause of visual loss in patients with optic nerve drusen is non-arteritic anterior ischemic optic neuropathy (NAION), which is reported to appear at a much younger age in patients with optic nerve drusen compared to patients with NAION unassociated with this pathology [**29**]. 

Another cause of vision loss in patients with optic nerve drusen is vascular occlusion, such as central and branch retinal artery occlusion and central retinal vein occlusion [**30**-**33**]. 

Hemorrhagic complications were also reported to appear in association with optic nerve drusen [**34**,**35**]. They were divided into three categories: small hemorrhages to the optic disc, hemorrhage extended to the vitreous and deep peripapillary hemorrhage extended under the retina [**36**]. Hemorrhagic complications were reported to have good visual prognosis [**5**,**37**] of most concern being those associated with choroidal neovascular membranes and macular involvement that can present with loss of central visual acuity. 

Choroidal neovascular membranes are complications that appear more often in children with optic nerve drusen, compared to adult patients. They are typically located in the peripapillary region and associated with a good visual acuity, in the absence of submacular fluid or hemorrhage [**38**,**39**]. 

In the case of the presented patient, the visual acuity at follow-up after 1 year was 20/ 20 in both eyes, despite the severe visual field loss especially in the left eye.

However, in this case, the patient’s prognosis on long term is uncertain. The high degree of structural and functional alterations of the optic nerve, especially in the left eye, provides a doubtful prognosis regarding the evolution, with high risk of irreversible loss of vision at a very young age.

**Financial Disclosures**

None of the authors has any financial or proprietary interests to disclose.
